# Women in a situation of homelessness and violence: a single-case study using the photo-elicitation technique

**DOI:** 10.1186/s12905-021-01353-x

**Published:** 2021-05-22

**Authors:** Clara Isabel Posada-Abadía, Carolina Marín-Martín, Cristina Oter-Quintana, María Teresa González-Gil

**Affiliations:** 1 Alcorcón Senior Residence, Madrid Social Care Agency, Madrid, Spain; 2 Department of Personality, Evaluation, and Clinical Psychology, Complutense University of Madrid, Madrid, Spain; 3 Nursing Department, Faculty of Medicine, Autonomous University of Madrid, Madrid, Spain

**Keywords:** Gender-based violence, Homeless persons, Adaptation, Psychological, Single-case study

## Abstract

**Background:**

Violence against women places them in a vulnerable position with regard to homelessness. Although sometimes invisible, women’s homelessness is a complex reality shrouded in dramatic biographies that should be sensitively addressed to avoid revictimization.

**Methods:**

With the aim of understanding the chaotic discourse of homeless women’s experiences of violence, a qualitative single-case study was conducted using the photo-elicitation technique. Data were analyzed in accordance with grounded theory.

**Results:**

The participant’s discourse could be summarized in the following categories: “Living in a spiral of violence”, “Confronting vulnerability and violence”, “Being a strong woman”, “New family networks”, “Re-building mother–child relationships”, and “Nurturing spiritual wellbeing”.

**Conclusions:**

Supporting homelessness women requires an approach that focuses on the prevention of re-victimization and the consequences of violence in terms of physical and mental health. Shelters are spaces of care for recovery and represent referential elements for the re-construction of self.

## Background

There are processes and situations that define women’s life paths and place them in extremely vulnerable scenarios. An example of this is homelessness. In recent decades, the study of the phenomenon of female homelessness has made it possible to ascertain the relevance of any form of violence against women in the lives of homeless women [1–3]. As adults, domestic violence may lead them to homelessness as they leave their homes to escape abuse [4, 5]. In other cases, when women are unable to access housing, they are forced to endure abuse and violence [6]. In addition, women who remain on the streets are regular victims of rape, sexual assault, robbery, insults, and threats. Shelters for homeless people are not safe spaces for them, as they are then at risk of being physically or sexually assaulted by other residents [7].

Even though being a victim of certain forms of violence, such as intimate partner violence, makes it possible for women to access specific housing resources, sometimes women’s biographies involve victimization accounts of certain types of “violence” for which there is a lack of a specific network of care. In other instances, their experiences of violence may remain invisible in the eyes of professionals [8], which is when their homelessness is seen as the primary problem and they are thus housed in specific residential facilities for this population.

Homelessness has emerged as a growing concern in a number of countries, irrespective of the continent or the per capita income of cities, suggesting that the phenomenon may be a more structural and/or social problem. For instance, in Madrid, Spain, the most recent nightly survey of homeless people identified 2998 individuals living in shelters, shelters for immigrants or social housing, or sleeping rough. Of those 2998 individuals, 11.2% were women. According to the same survey, 55.1% of homeless people said that they had been victims of some type of violence on the street. Gender-based violence was mentioned by 2.3% of the women as one of the main reasons for sleeping on the street, by 3% in the case of women living in a shelter, and by 7.4% of women living in social housing [9].

A number of studies report the role of structural and interpersonal gender-based violence in the trajectories of homeless women [10–12] and the relevance of gender-based violence in their pathways to homelessness. Understanding and looking deeper into biographies shaped by violence and the effects of said violence on the different aspects of the individual is thus extremely relevant and necessary for the design of policies and strategies to be able to meet demands for care. However, it is important for research to be conducted in a sensitive manner to avoid revictimizing women and for it to promote their empowerment using a trauma-informed approach to research [13, 14] and the emancipatory nursing praxis (ENP) as a nursing theory of social justice.

In line with the considerations set out above, this case study aims to unravel the chaotic discourse of a suffering woman who is repeatedly subjected to gender-based violence. Furthermore, on a methodological level, this study aims to demonstrate the experience of working with photo-elicitation [15], a tool with great potential when it comes to guiding and facilitating discourse production in individuals in a situation of extreme vulnerability. This technique offers us the possibility to facilitate the production of meaningfully rich data while empowering participants and keeping them safe from revictimization.

## Methods

### Contextualization of the case study

This case study is part of a larger study that aims to determine how homeless women perceive the violence they are exposed to and/or have experienced. To this end, a qualitative study was conducted under the theoretical proposal of symbolic interactionism and the methodological approach of grounded theory [16].

Maria (her name has been changed for anonymity) was recruited in the context of an observation program conducted using in-depth interviews in different shelters belonging to the municipal resource network of the Madrid City Council, Spain. Her account was identified as especially meaningful among the experiences of the 20 homeless women who were interviewed, exhibiting experiential characteristics relevant to the understanding of the phenomenon under study. It is for this reason that we set out to work from a single case study approach of an intrinsic nature.

Case studies focus on the uniqueness and complexity of a single case, gaining an in-depth understanding of it. Qualitative case studies are thus characterized by their intense nature, providing a deep, dense, and holistic description of a specific phenomenon from a naturalistic perspective.

In particular, we chose to conduct a unique case study of an instrumental nature, that is, the study of a critical or revealing case with its own suggestive characteristics that illustrates an exemplary situation. Our objective and reasons for doing so were, beyond simply understanding this individual’s particular situation, to contribute to the understanding of a broader phenomenon and to refine theoretical proposals [17].

### The participant

One of twenty women interviewed, Maria provided highly valuable data to clarify and shed light on certain aspects, key for the overall understanding of the adaptive processes of homeless women who are victims of violence and their struggle to move on and become highly resilient survivors. Maria is a middle-aged woman immigrant with primary education who arrived in Spain fleeing a situation of domestic violence in which she had been suffering since childhood, leaving her two children in her country of birth. Since her arrival in Spain, she has gone through different scenarios and situations of gender-based violence that have led her to homelessness. Despite the consequences that the experiences of violence had on her physical and mental health, Maria was able to develop coping strategies to give meaning to her life and keep her hope alive.

### Data collection

Data on Maria’s narrative were collected in different stages. The first stage consisted of conducting an in-depth interview in parallel with the interviews conducted with other participants in the study. An interview script was developed as a supporting element for the researcher (main author) (Table 1). The dynamic interview questions were designed based on the objectives, and the specific research questions were adapted to the participant’s level of understanding. Special attention was also paid to aspects related to the amplitude versus the specificity of the questions and the sensitivity and intimate nature of their content. The interview had a duration of 100 min and was recorded on audiotape for subsequent analysis with Maria’s permission.

**Table 1:** Script for the in-depth interview

Objectives	Dynamic questions
To analyze homeless women’s perception of their experience of intimate partner violence and/or violence from men in their environment	What has your history of homelessness been like? How did you come to find yourself in this situation? To what extent do you think your experiences of violence might have influenced your homelessness?
To identify adaptation or coping strategies generated throughout their experience of homelessness	How have you coped with the different difficult situations and experiences you have gone through? What things have enabled you to be strong in the face of adversity and suffering?
To identify internal elements or external barriers that have an impact on the continuity of homelessness	What difficulties do you consider that, on a personal level, limit your chances of escaping from homelessness? What barriers do you think your environment puts in the way of escaping from homelessness?
To analyze the interrelationships between women’s perceptions of the causes and consequences of the violence they have suffered and their reinterpretations	How do you understand violence? How do you understand violence against women? How do you think violence has affected your life as a woman since you were a child until now? How do you relate to violence? How do you deal with violence?
To identify their demands for care to guide the planning of woman-centered interventions	To what extent do you feel that the support resources you have received throughout your life match what you needed at the time? What help would you have liked to have received to avoid becoming homeless?

*Source*: authors' own creation

During data analysis, some meaningful elements emerged, inviting us to return to the field to delve deeper into and explore nuances, attributes and characteristics. One of these core elements that shaped Maria’s experience was artistic creation as an expressive resource and as a tool to work on coping and resilience. We met with Maria again to invite her to participate in our proposal to collect data on artistic activities, which were important in providing her with motivation.

Specifically, she was asked to continue to elaborate on her experiences through photo-elicitation. In this methodological proposal, first described by Collier in 1957 in the field of visual anthropology, photographic images are to be used as an evocative and provocative element of the informant’s experience, stimulating deep emotional aspects of their experience and rescuing visual metaphors as expressive and discursive elements of great potential [15–17]. In addition, Wang and Burris [18], allude to this technique with the term “photovoice” in the context of community research. These authors understand that the potential of this technique lies in giving people a voice through photography in what constitutes a creative and empowering process for them. Through photovoice, the individual identifies, captures, and visually represents aspects of their environment and daily experiences using photography, which then makes it possible to analyze the problems, strengths, and ups and downs of daily life through critical and reflective dialogue.

Previous studies have reported different experiences in the field where photography has been used as a provocative element in the sensitive discourse of individuals in particularly vulnerable situations, such as those of individuals with mental health problems [19], women who are victims of gender-based violence [20] and homeless women [21].

In this second meeting, Maria was told about the photo-elicitation technique, and she chose to use the camera on her own cell phone to take the pictures. It was explained to her that she could take pictures of things, objects, actions, places, or people (with their consent and without taking pictures or capturing elements that might reveal the person’s identity) that she considered significant in relation to her experience of recovery, coping, and resilience. She was also asked to write a brief commentary on each of the photographs, briefly summarizing why she chose to take that photo and explaining its significance. Maria took the photographs over a period of four weeks, which consisted of a total of 24 pictures. She decided to print them herself and write her reflections on the back in her own handwriting. Once the task was completed, the principal investigator collected the iconographic material to prepare the subsequent discursive meeting. In order to prepare this second interview using the photographs as an evocative element, a script was made using one of the photographs as an example (Table 2). The script was designed by two researchers individually, who agreed on a single proposal based on 14 of the 24 pictures taken by the participant (Table 3).

**Table 2:** Preparation process for the second interview using photo-elicitation

**Photograph**
Back of the photograph: “What I like best is the arts room. It’s killer for my recovery. I feel inspired and very happy, totally relaxed.”
**Description**
The core element of the photograph depicts the standing figure of a strongly built middle-aged woman, dressed in simple summer clothes, a blue tank top, shorts and sneakers, with a backpack and loose hair. She poses for the camera inviting us to contemplate two artistic works in what looks like an exhibition room; one of them is hanging on the wall at the back, in a wooden frame. The other one is next to the woman on an easel, where four handprints can be seen, two in the upper half of the painting in red and the other two in the lower half in black. The woman seems to be very familiar with and have an intimate knowledge of these creations
**Interview questions**
How do you see art contributing to your own recovery process?
How do you feel when you create?
What do you understand by inspiration?
What do you understand by a recovery process?

*Source*: authors' own creation. The image included in the table has been created by the participant in the context of the photo-elicitation

**Table 3:**
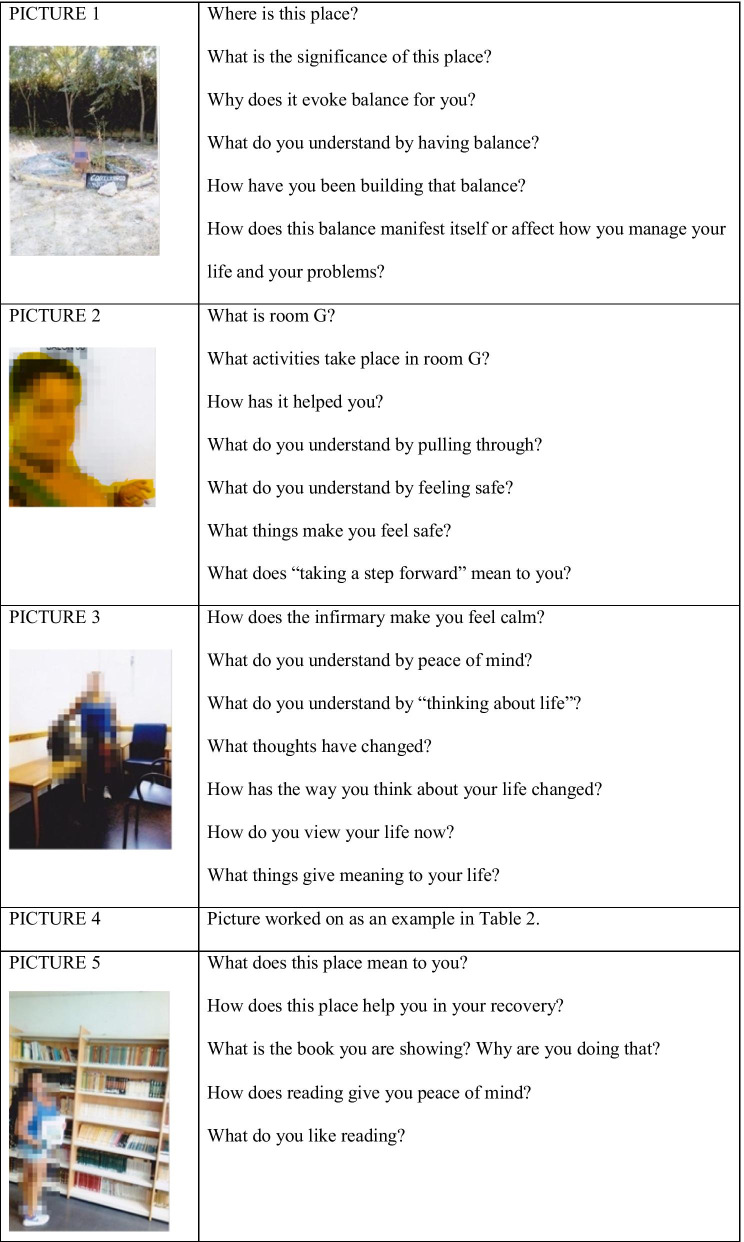

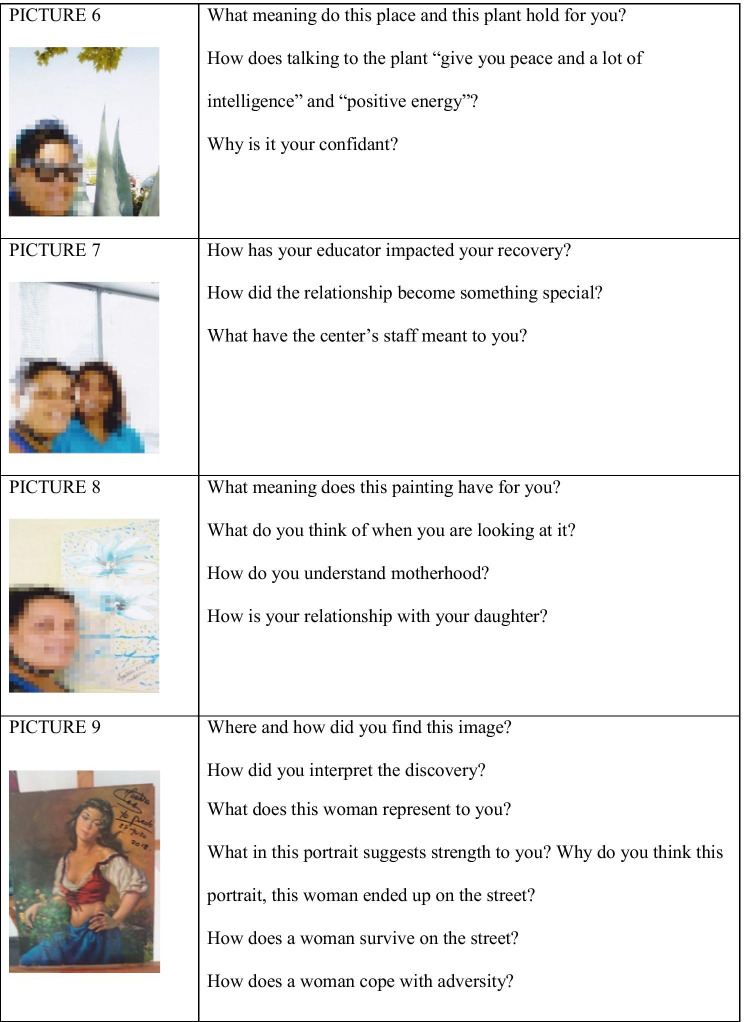

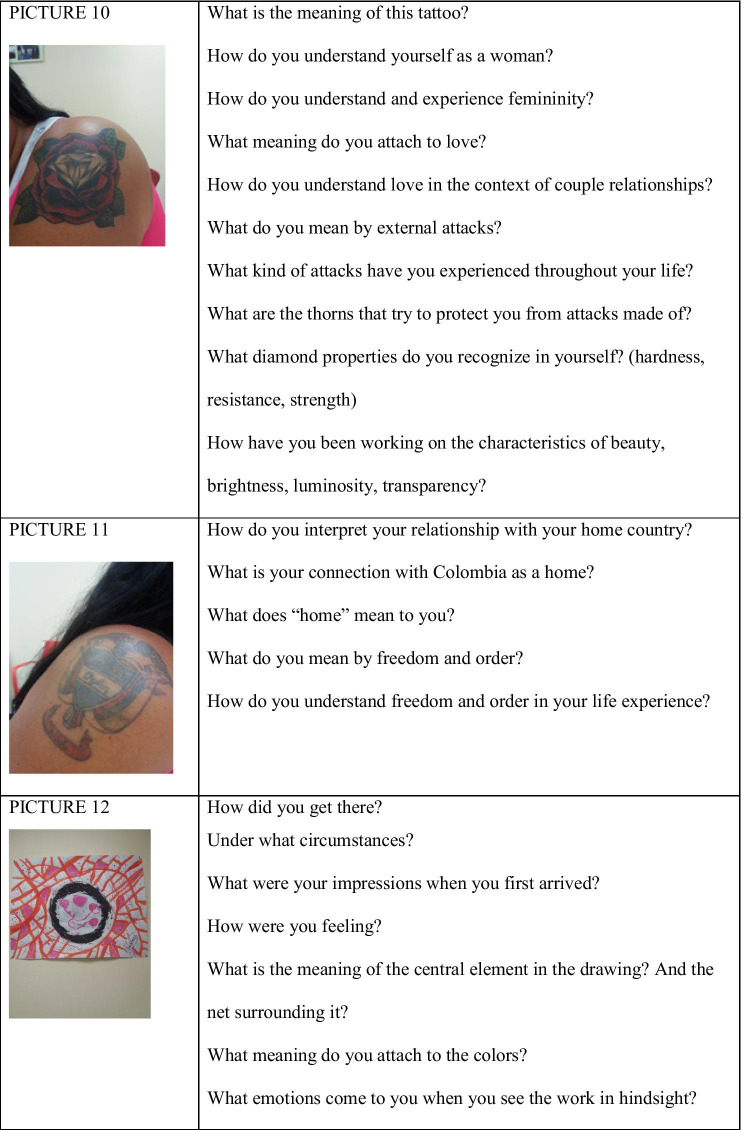

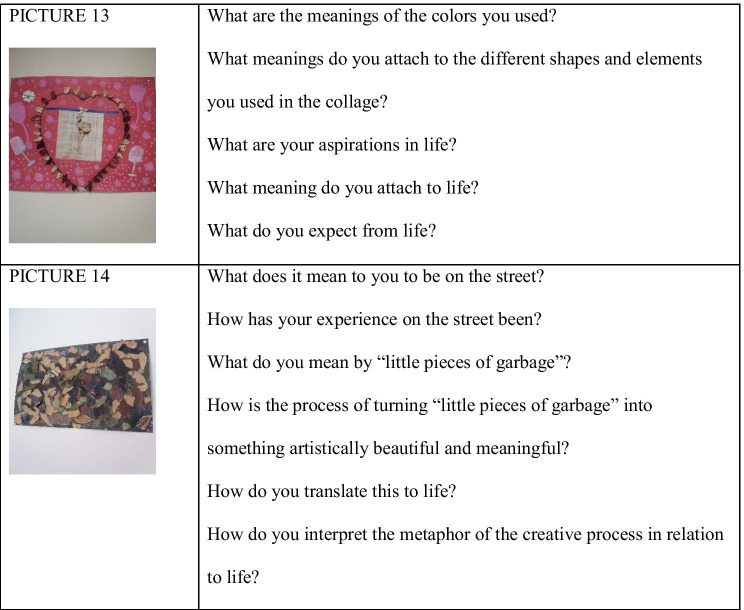
Script for the photo-elicitation interview

*Source*: authors' own creation. All images included in the table have been created by the participant in the context of the photo-elicitation

The interview was conducted in a meeting room of the shelter itself by the two researchers (main author and corresponding author) and had a duration of 100 min. During the interview, the interviewers sat on either side of Maria, keeping a distance of about 20 inches from her, and without any other elements in between (at a round table). The researcher was to show the photographs in the order suggested by the participant’s discourse, introducing the questions as appropriate. This task required a certain level of concentration and capacity of interrelation between the evocative resources and the evoked discourse. The second interviewer, on the other hand, focused on registering significant discursive aspects (as field notes) and introducing clarifying and explanatory questions in this respect. The interview was recorded on audiotape and transcribed in its entirety for subsequent analysis.

### Analysis

The analysis of the discourse produced during the first in-depth interview was performed by the main author following the grounded theory proposal [22], that is, an open coding of the interview was performed by identifying significant elements, the meaning of which, in relation to the study objectives, was condensed by means of free codes and in-vivo codes. Each of these codes was defined to form a glossary of codes. Some of these comments on the codes gave rise to incipient interpretative memos when compared to the analysis of interviews with other women. Subsequently, all the codes identified were interrelated in the form of a network in order to build a guide map to further deepen our understanding of Maria’s experience. This first phase of coding as assisted by Atlas.ti software (version 8.0).

The analysis of the photographs and the discourse produced during the interview was performed in different phases, as shown in Table 4, by main and corresponding authors. Firstly, an iconographic analysis of the images was carried out from the point of view of the manifest content, that is, from an essentially descriptive perspective of the element photographed [16]. This description was then linked to the written reflections written by Maria on the back of the picture and the discourse she produced during the interview with the picture in question. Once the materials had been interrelated, we proceeded to work on the open coding, taking as a reference the previously prepared glossary of codes, incorporating new codes and redefining others that were pre-existing. Codes were regrouped to create semantic groups that, little by little, gave shape to those categories that summarizes Maria’s process of coping, revealing the latent content of her discourse [16].

**Table 4:** Iconographic and interview analysis

**Photograph**
Back of the photograph: “When I was painting this picture, I was thinking of my daughter”
**Description (manifest content)**
The image is of a rectangular work of art, hanging on a light green wall. It is evident that it has been hand-painted with a paintbrush. There are two equally-sized flowers which occupy more than half of the work, one above the other, both colored with blends of blue and white, and with thin branches of the stems crossing each other, and with irregularly-shaped sky blue horizontally-oriented strokes. The background is grey with sky blue and yellow dots
**Discourse (interview)**
“They’re my two kids. Yes, C and V yes, that’s right, I see them as transparent and I can’t have them, I don’t know what to do! And that’s what it is, a pain I bear with me.”
“As a mother, a mother in pain, I am in pain because I cannot have them, nor my grandchildren, nor kiss them, nor play with them, nor tell my children [anything]; things like that are hard and I think I will go to the grave without being with my children… but I can’t… if that happens to me I’d be defeated.”
“The girl has disengaged from me, and the boy just the same! […] … This is very hard! I mean, she has a different mindset in another culture; of course, it’s been many years now… I haven’t enjoyed anything [of them] nor have I enjoyed [motherhood]. They are the children that have been ripped out! They were ripped out of me by the people who brought me here… […]”
“My children weren’t the fruit of love, they were fruit of rape […]”
**Analysis (Latent content)**
**Code: “Unfulfilled motherhood”.** It refers to the frustration of not having played the mother’s role from the participant’s perspective (providing protection, building emotional ties). This failure to provide maternal care is interpreted by her as a vital failure that generates intense suffering
**Code: “Ripped-out children”.** It refers to the violent separation from children. This separation is a consequence of homelessness and is characterized by an abrupt and forcible separation. The term “ripped out” suggests the notion that children are part of the participant’s identity as a mother. Separation is conceived not only as a physical separation, but also as a separation that leads to an acculturation of her children into their new hosting environment (cultural separation in relation to values, beliefs, ideas, customs, etc.)

*Source*: authors' own creation. The image included in the table has been created by the participant in the context of the photo-elicitation

### Ethical considerations

This study was approved by the Clinical Research Ethics Committee of Autonomous University of Madrid, Spain, under file number CEI-84-1555. Maria’s decision regarding whether or not to participate in the study was respected at all times and she was provided with all the information regarding the objectives of the research and the purpose, conditions, and possible benefits of her participation in it by means of an informed written consent form. Maria’s privacy and the confidentiality of her data were safeguarded by assigning codes and fictitious names to the individuals mentioned in her discourse. The provided participant name is fictitious and other possible in identifiers such as age and country of origin have been avoided. In addition, iconographic data have been pixelated guaranteeing participant anonymity.

Throughout the data collection process, special care was also taken to manage the emotional vulnerability of the participant through constant reflexivity and flexibility [23], by maintaining a sensitive attitude during the interview (using active listening, empathy) aimed at perceiving painful aspects and generating confidence and calm. In addition, the interview script questions, developed by themes and dynamic questions, were ordered using the “exploratory funnel” strategy, starting with questions of a more general nature and ending with the more specific and sensitive ones [24], supported by recovery models such as the “Tidal model”, by Phil Barker and Poppy Buchanan-Barker [25] and the “Self-transcendence” model, by Pamela Reeds [26].

### Quality criteria

Different strategies were considered to ensure the internal validity of the findings in terms of reliability and verifiability. Thus, during the research process, we worked on triangulating the researchers, both for the planning of the interviews and for the analysis of the data. In addition, study reports have been periodically produced and have been reviewed by an external expert. His recommendations, comments, and questions have served to delve deeper into some key aspects for their clarification.

Once the data had been analyzed and the report on the findings had been prepared, it was presented to Maria, so that she could read it and judge how her experience was reflected in it.

A sustained effort has been made to provide a detailed description of each of the processes undertaken in order to ensure the transparency of the research process and its methodological rigor.

Finally, a critical and reflective attitude has been maintained at all times by the researchers as a result of their commitment to considering themselves to be research tools [23, 27].

## Results

Maria’s experience has allowed her to provide an example of and illustrate very concrete and significant aspects of the experience of gender-based violence, homelessness, and the process of coping with vulnerability as a homeless woman. Her discourse may be summarized in the categories explained and illustrated below:

### Living in a spiral of violence

Gender-based violence in its different forms is present throughout Maria’s biography. Maria was born and lived her childhood and adolescence in a dysfunctional family environment where family violence was a part of everyday life. In her daily life, the maternal figure is blurred, without any female figure representing a reference point. She refers to her mother as both a recipient and giver of violence, fulfilling roles of submission and domination at the same time, maintaining power relationships that, in themselves, structure, normalize, and perpetuate violent relationships. Maria grew up in a hostile environment where she was socialized in a culture of abuse and violence and internalizes some attributes and characteristics of the female gender role that are very much tainted and conditioned by these values and beliefs. Maria grew up in the shadow of a negligent upbringing model, with no emotional ties, attachment relationships, or solid network to support her.

Then... among the members of my family everything was dirty because of drug trafficking, cocaine from drug trafficking; I was already being abused by my father, by my mother […]. I was sexually abused in my childhood repeatedly by the lovers my mother brought home! She’d sneak them into the house behind my father’s back... I remember that they used to touch my legs, my privates... up here as well (touching her breasts) [...].I was sexually abused many times in my childhood [...]. I do not have one nice memory, everything [I remember] is awful!

During adolescence and early adulthood, as a consequence of this culture of violence against women, Maria was subjected to sexual violence, raped by people in her family environment, resulting in two unwanted pregnancies. It is then that she began to experience first-hand the objectification of women. Sexual violence engulfs her everyday life and pushes aside the notion of healthy interpersonal relationships to an unreachable place. Relationships were constructed by and identified with violence; violence became, therefore, Maria’s way of relating to her environment.

[…] Neither was adolescence. I was raped and had two children who are not the fruit of love, but of rape... So what life have I had? An awful life, a hard life! […]. I was sexually abused, sexually abused by uncles, nephews, cousins […].

Maria then decided to put distance between herself and the violence and flee from this scenario, leaving her two very young children behind. During her escape, she unknowingly entered a network of women traffickers where gender-based violence against women made her an “item of merchandise”. For almost ten years, she was sexually exploited and, at the same time, she was forced to use narcotics to be able to keep up with carrying out sex work 24 h a day, due to being in an intensive and alienating production system. It was at this stage that sexual violence became linked to economic violence. Violence became the only element that linked her to her surroundings, trapping her in a dead-end spiral where violence feeds on violence, with the exercise of violence being the only way to survive this situation.

[…] I was pimped [...]. I was sold off for two horses [...]. I was practically trafficked, [that was] human trafficking, sex slavery. I didn’t choose that life, it chose me, I didn’t choose it.

At this moment, Maria says that she lost her sense of self. Her identity is blurred to adopt blurred, indecipherable, unrecognizable strokes. With nothing left to lose, but still with the hope of regaining her identity and her lost life, Maria decided to put an end to this situation and escaped again.

Leaving the previous situation meant entering a situation of structural violence. In spite of having been able to report the situation she has experienced, Maria found herself without the necessary resources to support herself in her situation of real vulnerability, which is when her experience of homelessness and begging started. The streets became a place to live in and from which to live and, little by little, begging was replaced by theft, which eventually led to another scenario of violence: prison. Maria’s discourse invites us once again to reflect on the structural violence (administrative-legislative) that relocated her to a new scenario where freedom was taken away from her.

[…] Once on the streets, I started serving jail time, all because of petty theft, nothing violent [...]. It was all because of stealing for my sustenance, that’s when I became a criminal.

In prison, Maria found a hostile environment where symbolic violence was exercised by both the institution and her peers. According to her discourse, this last experience was the straw that broke the camel’s back, overwhelmed her, and accelerated a series of health-illness processes in her physical and mental spheres. Far from being able to be considered, to a greater or lesser extent, a rehabilitative resource, prison ended up devastating Maria’s identity, stripping her of all traces of dignity, self-recognition, and hope. Maria’s entire life journey through violence manifests itself explicitly with signs and symptoms consistent with post-traumatic stress disorder.

After serving her sentence, Maria took to the streets, where she once again faced homelessness, this time without intrapersonal or interpersonal resources and in a situation of extreme vulnerability and social exclusion. It is at this point that she entered the social support network through the street services and began her life experience in the shelter.

[…] I was on the streets like a ‘loony’, for almost two and a half years... [...] ... stealing. I went to prison for three years... I’m screwed; I have post-traumatic stress, with bipolar disorder [...], many diseases... [...]... I’m celiac... [I have] problems with my breasts...

Some time after being in the shelter, after intense work and important advances in the recovery process, Maria met her partner with whom she built a life project outside the shelter, as a result of her interaction with her peers in the shelter and her search to establishing emotional bonds. This relationship is understood by Maria as a bid to build a full and meaningful life, with the loved one as the core element. However, intimate partner violence emerged to complete the spiral of gender-based violence and sent her back to the streets, forcing her to return to the shelter, to the comfort and support of what she understands as her only family.

[…] He approached me as a friend and I began to fall in love with him... I had never felt anything like that about anyone before... He asked me to leave [the shelter] and so we did... He had an income and we lived on it in a room... and we got married. […].Over time, he began to mistreat me, to insult me... shocking jealousy and from there to the arguments, fights... hitting... and after two years, one day he threw me out of the room and I was back on the street with my luggage...

### Confronting vulnerability and violence

Faced with a life full of violence and in a situation of extreme social vulnerability, Maria reflects on her strength and capacity to overcome obstacles. Different coping strategies are identified in her discourse such as: the redefinition of herself as a strong woman; the creation of new family networks; the strengthening of a maternal role in her own identity; and the nurturing of her spiritual wellbeing. The development of these strategies has allowed her to advance in her recovery process, that is, to advance in the redefinition of herself and in the recovery of her capacity of self-determination and ability to make decisions about her own life, so that she can achieve a sense of fulfillment, a sense of personal wellbeing and health, and of social involvement and good quality of life. Maria’s discourse, however, does not allow us to identify procedural aspects that clarify when these strategies were developed or what factors may have facilitated or promoted their development. Instead, her narrative, which is viewed through a certain retrospective lens, touches on these resources in terms of results. Nevertheless, one can sense a decisive event that marks a change in her path, which is her leaving the streets and taking refuge in the shelter.

[…] I think you have to have like a... how should I put it?! I can’t explain it, be myself! To be, to continue with what I have to do, not being involved in prostitution, drugs... [Now I feel] that I am kind of free, I am free in spirit [...], that I can do what I want: eat, paint, sleep; nobody harasses me, I’m not on drugs, I have my freedom, I can go to church, paint, be in the shelter; I can talk, it’s like… something that’s very beautiful, it’s something wonderful, something I don’t know how to explain; I am now kind of free from where I was for so long; I was pimped for many years, forced to be with various men, I was like that back then and now I am free from anyone telling me to do this or that; when they tell me something here they tell me nicely, and, to me, that means freedom, I am finally free.

### Being a strong woman

Maria understands herself as a strong woman, interpreting strength from the notion of vigor, vitality, drive, nerve, or energy. At the same time, in her discourse one can glimpse the idea of strength as a bastion or bulwark, that is, as a solid and strong space for defensive tasks. Without a doubt, the case of Maria metaphorically responds to the idea of a bastion within a defensive stronghold in the face of continued attacks on her person, the various forms of violence.

[…] I say I don’t know if I’m strong or if I’ve been touched by a star or... what has touched me? Tell me! […]. I found it (see Table 3, picture 9) around Príncipe Pio and I brought it and kept it in my room [...]. Ah, yes, how beautiful, I don’t know, this picture is impressive [...]. I see her as being very angelic, with a very angelic face, I see her as being natural [...], I consider her to be strong. In all that I do… here, for 24 years, I’ve kept holding on and I consider myself strong; I think I am strong; I don’t know if I may be strong to other people, but I think I am strong.

She recognizes a gift, a star, in this strength, and is aware of the other women who, having shared experiences similar to hers, have fallen by the wayside. She sees in feminine attributes and characteristics a great capacity for self-improvement and resilience with which she identifies and builds herself by rebuilding and strengthening her identity.

### New family networks

In her discourse, Maria refers to the creation of new support networks and the establishment of new bonds, which she understands as her new family.

Her process of re-affiliation through new social bonds became her main material and emotional resource for her subsistence and adaptation to daily life. The establishment of networks not only with other homeless individuals, but also with personnel from the shelter, people from neighboring areas, religious institutions, and support associations, allows her to improve her self-esteem and sense of belonging by reducing her isolation and feelings of loneliness.

[…] My family couldn’t be bothered to look for me or anything, I pulled through by myself and with the help of the shelter. Your family is where you live! This is my family!

The shelter thus became her reference point, her emotional family, her home. Therefore, her home is interpreted as a relational space that provides her with protection against the dangers of the streets. Maria refers to the shelter as her formal adaptive psychosocial support network, where she has established special bonds with the center’s staff and other users. Interaction with what surrounds her and those around her allows her to build new meanings around the reality she has come to live, by sharing beliefs and values, ways of doing and understanding things, which link her to the shelter, even to the point of considering it as “her home”. The shelter, and her belonging to it, takes on meaning by becoming that which she never had at her side, her family. In addition, Maria also makes the space her own by decorating rooms with her works, which shows how our home shapes our identity and vice versa.

Freedom is what I have always longed for, what I am looking for; I came here from my country looking for that freedom, to get out of the horror of my family..., I came here to bring order to my life, to see what a home really is, in spite of everything, and all this is the next best thing... paintings of mine that I have been doing as therapy to get out of my system everything that I had inside, all that anger, fear, pain, and I wear it like a tattoo so that I don’t forget my country, to which I will never return, not even to meet my children.

### Re-building mother–child relationships

For Maria, the fact that she missed out on raising her children generates frustration and pain. Her childhood experiences of lack of love and care became learned behaviors from her mother’s role as a model of insecurity in her relationships and, consequently, deficiencies in her supporting role for her children. The loss of all contact with them over many years leads her to work on redefining mother–child relationships and maternal roles in order to justify a vital motivation and overcome “the pain of an unfulfilled role”. She alludes to the fact that it is violence which has deprived her of the opportunity to become a mother using the expression “the children that have been ripped out”. Maria feels motivated and hopes to establish emotional ties with her children and now grandchildren; to rebuild the emotional bond that was broken by time and space after years of separation.

I dream of all of us being together, meeting and touching my children. I haven't touched them for years, since I came here... My children were babies when I left them. Now they each have their own life and I have mine. […] I have so many aspirations in life… I aspire to many things... to get out of these rough patches. I would like to have my children by my side, to aspire to see them, to have them and be with my family... It is one of my main aspirations, to meet my three grandchildren... but it is impossible, I do not know why this has happened to me; it is impossible, the girl has another culture and the boy too; they already have different lives. [...] They are the children that have been ripped out, they were ripped out of me.

### Nurturing spiritual wellbeing

Nurturing spiritual wellbeing by “seeking balance” refers to Maria’s willingness to experience and integrate the meaning and purpose of life by connecting with the self, others, art, music, literature, nature, or a higher power than oneself, which can be strengthened. In her discourse, Maria alludes to the search for this wellbeing through various aesthetic and creative resources. Thus, she refers to having found in artistic creation processes a way to represent and externalize her pain and suffering. Creative processes thus became a key element in her recovery process, with great therapeutic potential. Writing, painting and photography are understood as projective elements that allow her to explore her emotions, perceptions, and behaviors from within her freedom, providing opportunities for self-knowledge and personal growth.

When I paint I feel very happy, I forget about all my problems, my hand doesn't shake, I forget about terrible things [...]. I like to paint, to be calm, and to get out everything I have inside.

This intrapersonal knowledge opens up one’s consciousness to working on the spiritual dimension and encourages the development of new strategies and resources for self-transcendence. In Maria’s case, moreover, religious beliefs and practices (reading the Bible, praying, volunteering in a church) coexist to enhance and promote this spiritual work, which gives her self-confidence and hope.

(See Table 3, picture 1) I’d sit there and say: My God, I must have a balance! When I leave here I have to be stable and not get into drugs, alcohol, prostitution... God give me that strength!

## Discussion

Maria’s case study had a twofold purpose: to understand the experience of a homeless woman who was a victim of gender-based violence, which was considered to be a particularly representative case because of her ability to cope with adversity; and to generate a space for free research where the participant and the researchers could test new discursive codes to explore the experiences she had lived.

Through photo-elicitation it has been possible to share a genuinely different experience, capable of expanding discursive possibilities when with words alone it is difficult to support the narrative of experiences because of their harshness and situations of extreme vulnerability [28, 29]. Nevertheless, it is necessary to point out that this technique has required effort, time, capacity for introspection, as well as certain skills in the use of technical means from Maria. In addition, this technique has been expensive for the researchers themselves, since it has required a complex process of planning, monitoring, and analysis.

Through this case study, Maria was able to describe numerous situations throughout her biography where violence (in all its forms and aspects) was present in various situations occurring consecutively, from childhood to mature adulthood. Homeless women are a very heterogeneous group and, as a result, so are the paths that lead them to homelessness as well as their later trajectories. However, there are circumstances, such as violence, that put them in a particularly vulnerable situation. Violence in the family environment during childhood is pointed out in numerous studies as a common factor for many of these women [30].

These experiences of violence have resulted in serious physical and mental health problems. Consistent with Maria's experience, the evidence indicates that post-traumatic stress disorder is the most frequently developed mental health disorder among victims of various forms of emotional, physical and sexual violence in both childhood and adulthood [3] and that this condition strongly influences the development of coping styles [31, 32]. Cumulative exposure to stressful events, mostly related to violence (with special emphasis on domestic violence and childhood sexual abuse), is also a factor related to substance abuse. Homeless women refer to the fact that, sometimes, substance abuse is closely linked to the relational environment in which they live. On other occasions, there is a therapeutic justification for substance abuse, such as the self-management of anxiety and emotional distress [33].

In addition, we see how Maria has been in and out of homelessness at different times in her life. In line with this, Broll and Huey [34] conclude that homelessness tends to be cyclical, with women entering and leaving this situation. The results of the monitoring of a cohort of 269 homeless women suggest that this trend is greater among women who have experienced multiple forms of violence (e.g. physical or sexual abuse) during different stages in life (childhood and adulthood).

In spite of the circumstances experienced and their impact on women’s physical and mental health, women still seek and find motivation to continue to fight and move forward [30]. The latter can also be clearly seen in Maria’s narrative. The identification and exploitation of internal resources, the strength she refers to, hint at coping from an adaptive approach, which is grounded in an exercise of restructuring and redefining one’s own self and one’s support environment [35]. The way in which she describes herself is from her encounters with others; it is from relationships that the individual is identified and reconstructed [36], tracing the path to move forward [37].

The lack of a family and affective ties makes Maria transform the shelter into her “home” by generating new networks that allow her social ties to be re-affiliated, thus reducing isolation and the feeling of loneliness. This experience is consistent with the findings of Groton and Radey [38], who mention the loss of primary social networks such as the family and violence in the relationships within one’s social networks as key elements leading to homelessness. They also conclude that building new social networks is a powerful coping mechanism and that willingness to help peers generates a strong sense of wellbeing. Other studies also mention that living together in shelters enhances empowerment and self-esteem [39] and individuals interpret their experiences there as positive, particularly with respect to staff. In general, the shelter is a place of respite, providing safety and warmth after having been abused and socially isolated [40].

Domestic violence in childhood and intimate partner violence are significant psychosocial contextual factors that affect the regulation of emotions, fostering the development of post-traumatic stress and compromising future parenting [41]. In addition, learned helplessness [42] and the distorted maternal role model, with no reference figures or child attachment, shape the individual’s future ability to provide safe parenting [43], defining a profile of vulnerability in one’s role as a mother. Similarly, maternal identity is compromised by not fulfilling one’s role as a mother [44]. The restoration of mother–child ties would not only allow her to learn about the fate of her relatives and children, but also to heal wounds and rebuild her relationship with them. These findings reinforce the importance of supporting maternal mental health and facilitating processes that allow women exposed to violence to recover [45].

Moreover, cultivating spirituality is another key aspect of recovery from trauma, focusing on oneself as a form of self-care, regaining control and empowerment [46] and God as a source of comfort and post-traumatic growth [47]. This echoes the findings of Ahuja et al., who conclude that levels of subjective wellbeing are lower among homeless people (“unhoused people”) compared to housed participants, especially in dimensions such as social connectedness, negative emotions, perceived stressors, and resilience. On the other hand, spirituality and religiosity are more developed among unhoused people, who experience a more pronounced need to search for meaning and connect with a higher being [48].

Finally, the exploration and expression of feelings through artistic creation, in the case of Maria, show that art therapy based on creative processes helps to improve the therapeutic relationship, restore balance, and channel emotions [49]. This finding demonstrates the importance of using art as therapy to improve self-esteem and provide more positive future prospects [50]. Creative processes facilitate the construction of escape spaces where one is able to “switch off”, to “change reality”, to “isolate oneself”, and they constitute a powerful strategy for the control of anxiety and emotional suffering. Through creative processes, emotions are identified, evoked, channeled, and transferred. At the same time, homeless people identify these spaces as safe and healing and as a tool for self-knowledge and for the transformation of their relationship with themselves. These are spaces that facilitate self-expression by recognizing oneself in one’s creations, exploring one’s own identity, and rediscovering facets of oneself that one did not think existed, but do exist [50]. The findings described in this study may have implications for both shelter policy and practice with respect to caring for homeless women, but also for addressing their mental health and social and family integration.

To conclude, it should be noted that the reality of the victimization of homeless women requires approaches along the lines of “trauma-informed care”, a philosophy of care that focuses on the recovery of women, taking into account their pathways of exposure to gender-based violence, emphasizing their physical and emotional safety, their recovery of self-control, their strengths versus their weaknesses or deficits, and, ultimately, strengthening their capacity for resilience and empowerment [51].

## Conclusions

Gender-based violence, in its different aspects and representations, is a constant in the trajectories of homeless women. Welcoming and supporting them requires an approach that focuses on the prevention of re-victimization, as well as on the consequences of this violence in terms of physical and mental health. Shelters emerge as spaces of care for recovery and represent referential elements for the re-construction of bonding relationships, the creation of supportive environments, and the meeting point of development spaces in freedom and the capacity for self-determination. Sensitive, trauma-informed research should be used to inform the support offered to homeless women as part of narrative advocacy-based care.

## Data Availability

The complete (de-identified) dataset generated and analysed during the current study can be made available from the corresponding author on reasonable request.

## Abbreviations

MSc: Magister Scientiae (Master of Science. Master Degree)
PhD: Philosophiæ doctor (Doctor. The highest University Degree)
PhD Candidate: Philosophiæ doctor candidate
Psyc: Psychologist
RN: Registered nurse
